# Embodied Cooperation to Promote Forgiving Interactions With Autonomous Machines

**DOI:** 10.3389/fnbot.2021.661603

**Published:** 2021-04-09

**Authors:** Jonathon S. Schofield, Marcus A. Battraw, Adam S. R. Parker, Patrick M. Pilarski, Jonathon W. Sensinger, Paul D. Marasco

**Affiliations:** ^1^Department of Mechanical and Aerospace Engineering, University of California, Davis, Davis, CA, United States; ^2^Department of Medicine, Faculty of Medicine and Dentistry, University of Alberta, Edmonton, AB, Canada; ^3^Department of Electrical and Computer Engineering, Institute of Biomedical Engineering, University of New Brunswick, Fredericton, NB, Canada; ^4^Department of Biomedical Engineering, Lerner Research Institute-Cleveland Clinic, Cleveland, OH, United States; ^5^Advanced Platform Technology Center, Louis Stokes Cleveland VA Medical Center, Cleveland, OH, United States

**Keywords:** embodiment, human–machine interaction, autonomous machine, bidirectional interface, perception, cooperation

## Abstract

During every waking moment, we must engage with our environments, the people around us, the tools we use, and even our own bodies to perform actions and achieve our intentions. There is a spectrum of control that we have over our surroundings that spans the extremes from full to negligible. When the outcomes of our actions do not align with our goals, we have a tremendous capacity to displace blame and frustration on external factors while forgiving ourselves. This is especially true when we cooperate with machines; they are rarely afforded the level of forgiveness we provide our bodies and often bear much of our blame. Yet, our brain readily engages with autonomous processes in controlling our bodies to coordinate complex patterns of muscle contractions, make postural adjustments, adapt to external perturbations, among many others. This acceptance of biological autonomy may provide avenues to promote more forgiving human-machine partnerships. In this perspectives paper, we argue that striving for machine embodiment is a pathway to achieving effective and forgiving human-machine relationships. We discuss the mechanisms that help us identify ourselves and our bodies as separate from our environments and we describe their roles in achieving embodied cooperation. Using a representative selection of examples in neurally interfaced prosthetic limbs and intelligent mechatronics, we describe techniques to engage these same mechanisms when designing autonomous systems and their potential bidirectional interfaces.

## Introduction

From smartphones to self-driving vehicles to advanced artificial limbs, cooperative machines are becoming increasingly integrated into our society. As they continue to grow in their level of sophistication and autonomy, so does the complexity of human-machine relationships. When engaging with technology, frustration is never far away and negative emotions may shape our disposition to using a technology (Klein et al., 2002). Sometimes these emotions are merited by the poor performance of the technology, but we often misjudge technologies and place unfair expectations on them (Jackson, 2002) simply because of the way they communicate with us. Like the way the glimmer of a smile or the touch of a hand can change our reception of hard news, the way that technologies interface with us is imperative to accepting their capabilities. As humans, we are quick to distinguish between ourselves and cooperating machines, and to blame them for errors (Serenko, 2007). However, the perception that our bodies and our actions are our own is incredibly malleable and this malleability provides a pathway to improved human-machine interactions. Our brains and our bodies host a variety of conscious and non-conscious perceptual mechanisms to perceive ourselves as separate from our environments, and these mechanisms may be targeted through bidirectional machine-interfaces. In doing so, we may assume ownership of cooperative machines and their collaborative actions to promote more forgiving interactions, a concept we call embodied cooperation.

## Our Actions and Our Biases

How do we know that we are “ourselves”; autonomous agents that have physical bodies, and act within an external environment? Although various forms of this age-old question have long been explored across disciplines including philosophy, phenomenology, psychology, and cognitive neuroscience; a single unifying theory of self-awareness has yet to be developed (Braun et al., 2018). Rather, there are several neurocognitive theories that hypothesize varying degrees of influence from the brain integrating multisensory information and internal representations of the body (Tsakiris, 2010; Braun et al., 2018). What is clear, is that our brains readily and constantly distinguish ourselves as separate from our environments, the tools we use, and the people around us. These distinctions of “self or other” shape our perceptions of nearly every action we perform.

There is a spectrum of control that we have over the outcomes of our actions that spans the extremes from full to negligible correlation. In between, our *perceived* role in an outcome is directly linked to the brain's distinction of self or other. As individuals, our locus of control describes the degree to which we believe that *we* control the events around us, as opposed to *external forces* (Rotter, 1966). There are many factors that may shape this perception including age, gender, and cultural differences (Strickland and Haley, 1980; Berry et al., 1992; Hovenkamp-Hermelink et al., 2019); however, there are inherent biases in our perceived control of events. The term self-serving bias describes the larger group behavior in which we tend to disproportionately credit ourselves for positive outcomes of actions while blaming negative outcomes on things beyond our control (Davis and Davis, 1972). This behavior has been observed in numerous contexts including competitive sports (Lau and Russell, 1980; Riess and Taylor, 1984; De Michele et al., 1998), perceptions of one's own employability (Furnham, 1982), and academic performance (McAllister, 1996), among many others (Gray and Silver, 1990; Sedikides et al., 1998; Farmer and Pecorino, 2002). Self-serving bias is highly relevant to human-machine interactions as people tend to not only blame technology for mistakes but are also less likely to attribute positive outcomes to machine-partners and even take credit for themselves (Friedman, 1995; Moon, 2003; You et al., 2011).

Autonomous machines have an even more troublesome relationship with self-serving biases. This behavior is observed during interactions with artificial intelligence (Vilaza et al., 2014) and amplified as the degree of machine autonomy increases (Serenko, 2007). Further, in the event of an inappropriate interaction, frustration and emotional consequences are never far away. Negative emotional states are linked to more extreme self-serving behaviors (Jahoda et al., 2006; Coleman, 2011) and frustrating interactions can leave users negatively disposed to technologies (Klein et al., 2002). Here, there is a difficult “blame cycle” in which systems of increasing autonomy receive increased blame for errors and these errors can promote negative emotional states that further reinforce the displacement of blame. Rather than blaming and becoming frustrated with our technological partners, we need to develop more forgiving relationships to break this blame cycle. As humans, we do have the capacity to form these forgiving relationships. For example, individuals are more inclined to assist a computer to complete a cooperative task if that same computer has previously assisted the user (Fogg and Nass, 1997). Further, Mirnig et al. performed a study in which participants were provided simple task instructions by an anthropomorphized social robot. Participants described the robot as more likable when minor non-task-related errors were made, suggesting that like perceptions of other humans, minor imperfections carry the potential of increasing likability (Mirnig et al., 2017). Therefore, we argue that natural human tendencies and biases also provide opportunities rather than just barriers to improve interactions with autonomous machines. We further suggest that if a technology (and/or its actions) can be perceived as belonging to the user, many of our existing biases may be flipped to the benefit of more effective and forgiving cooperation.

One might think that autonomous machines would be more easily accepted and forgiven, given that our brains are hardwired to cooperate with the autonomous processes in our own bodies. For instance, a single motor task may be achieved by nearly infinite combinations of joint motions and timings (Bernstein, 1967). To be completed without attending to every muscle's action, the central nervous system appears to rely on repertoires of autonomous movement patterns (Bizzi et al., 1991; Wolpert et al., 2001; Giszter and Hart, 2013). Although we feel in complete control of our limbs and bodies, when we move our bodies or manipulate objects, the specifics of those motions are executed through autonomous sensorimotor control loops outside of our conscious control. It is this biological-autonomous framework that cooperative machines should seek to engage. To do this, carefully constructed bidirectional interfaces may be employed. Like our biological bodies, these devices will need to consistently and accurately trigger a machine to perform cooperative actions while also returning relevant and temporally appropriate information to the user.

## The Mechanisms of Embodiment

**Figure 1:**
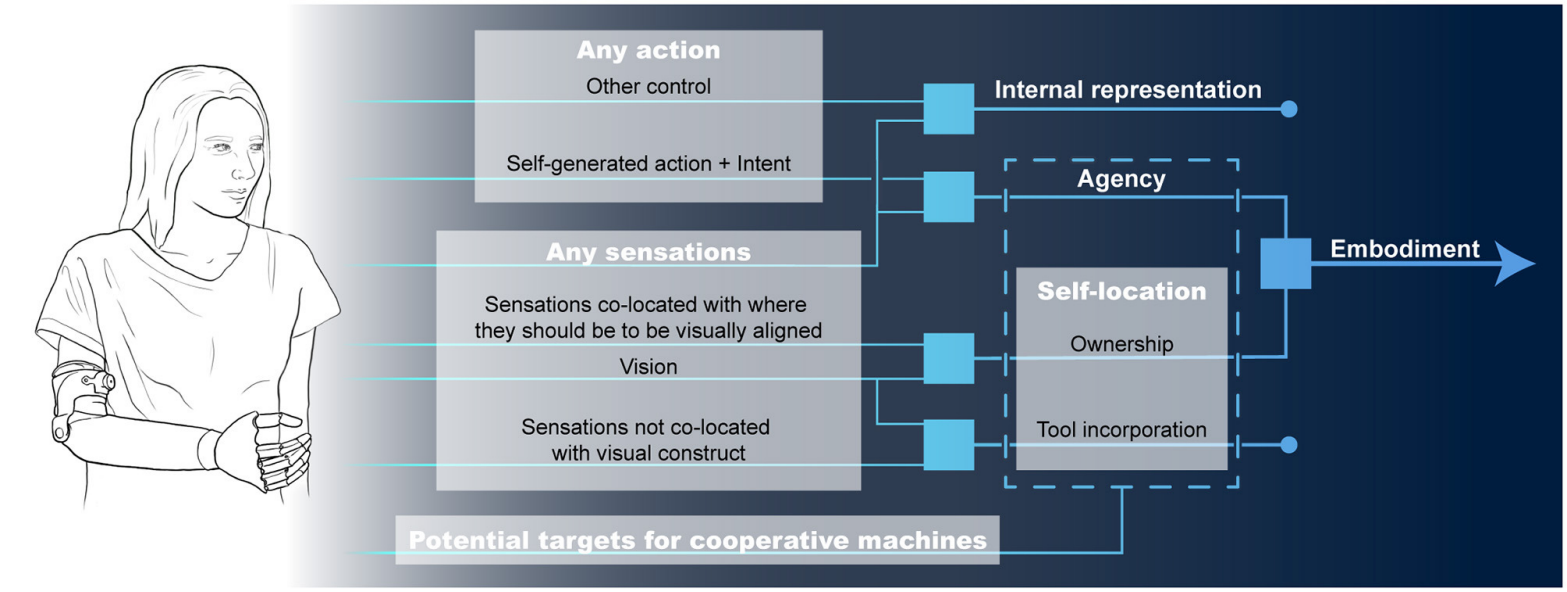
The pieces of embodiment and potential targets for cooperative human-machine interactions.

In nearly all interactions with cooperative machines, we perceive ourselves and our actions as separate from the machine. This distinction is a product of our sense of embodiment. Here, we adopt the definition of embodiment as the combined experiences of owning and controlling a body and its parts (Matamala-Gomez et al., 2019; Schettler et al., 2019). Embodiment emerges from the integration of our intentions, motor actions, and sensory outcomes (Braun et al., 2018; Schettler et al., 2019). More specifically, it integrates perceptions and mental constructs built around vision, cutaneous sensation, proprioception, interoception, motor control, and vestibular sensations (Maselli and Slater, 2013). The sense of embodiment is malleable and manipulating these channels can extend the perceived borders and capabilities of our bodies to include non-bodily objects and even cooperative machines (Botvinick and Cohen, 1998; Braun et al., 2018; Schettler et al., 2019). In this context, there are three key experiences that a machine may engage with, these are: (1) the sense of self-location, experienced as the volume in space where one feels their body is located; (2) the sense of ownership, the experience of something being a part of the body; (3) and the sense of agency, the experience of authoring the actions of one's body and the resulting sensory outcomes (De Vignemont, 2011; Kilteni et al., 2015). Figure 1 illustrates the relationship between actions, intentions, sensations, and the experiences that summate to the sense of embodiment. In this paper, we discuss varying degrees in which machines may engage these experiences to create a spectrum of perceptions that spans between operating a tool as an extra-personal extension of the body through the complete embodiment of a machine.

### Self-Location and Tool-Incorporation

In literature, the larger concept of embodiment is often confounded with tool-incorporation which is the extra-personal experience of operating a tool as an extension of the body (rather than a part of the body). Promoting tool-incorporation in autonomous machines may be achieved by simply providing appropriate sensory feedback to the user. In doing so, users may develop a keen awareness of the tool's physicality as the brain adapts its geometric representation of the body and surrounding workspace (peripersonal space) (Iriki et al., 1996; Schettler et al., 2019). For example, the haptic feedback provided through canes used by visually impaired individuals can promote tool-incorporation. This results in an expansion of peripersonal space and an acute awareness of the area around the cane's tip (Serino et al., 2007). Similar effects are observed in numerous tools spanning the complexities of a rake through an automobile (Iriki et al., 1996; Sposito et al., 2012; Moeller et al., 2016). However, at no point do these users perceive their tools as a part of their bodies as they do not engage all the key mechanisms of embodiment. Here, peripersonal space and the sense of self-location are closely linked and may be influenced by tool use (Noel et al., 2015). Further, tool use may even promote a sense of external-agency (described below). However, these tools do not provide visually collocated feedback, which is required to form a sense of ownership, the distinguishing factor in this case. Of further relevance to human-machine cooperations, there appears to be a link between tool-use proficiency and the changes in peripersonal space that accompany tool-incorporation (Sposito et al., 2012; Biggio et al., 2017). For instance, experienced drivers underestimate distances in front of their vehicles (Moeller et al., 2016), and skilled archers perceive their targets as larger (Lee et al., 2012). Therefore, further exploring tool-incorporation and how cooperative machines may engage the requisite sensorimotor mechanisms may be an important avenue to accelerating user proficiency.

### The Sense of Ownership

The sense of ownership is the experience of our body and body parts belonging to ourselves and describes the feeling of “mineness” that we experience (Braun et al., 2018). It is the feeling that is captured in statements such as “this is ‘my’ hand,” and it often occurs at the fringe of consciousness (De Vignemont, 2011; Braun et al., 2018). There is strong evidence suggesting the sense of ownership is a product of the integration of visual and (most commonly) tactile sensory channels (Botvinick and Cohen, 1998; Kilteni et al., 2015; Braun et al., 2018; Schettler et al., 2019). Much of our current understanding originates from the rubber hand illusion in which participants report experiencing a rubber hand as a part of their bodies with strategic manipulation of what they see and feel (Botvinick and Cohen, 1998). This well-known experimental paradigm demonstrates that our experience of body ownership is dynamic, adaptable, and is not constrained to our biological body parts. Not only does the rubber hand illusion influence participants' sense of ownership, but it also influences the sense of self-location. When participants are asked to close their eyes and point to the location of their hand, their estimates are typically shifted toward the rubber hand (Botvinick and Cohen, 1998; Tsakiris and Haggard, 2005). This finding suggests that the brain is updating its body representation at conscious and non-conscious levels, with other non-conscious temporary physiological changes being observed, including hand temperature (Moseley et al., 2008), touch and pain sensitivity (Folegatti et al., 2009; Fang et al., 2019), skin conductance (Ehrsson et al., 2007), and cortical excitability (Della Gatta et al., 2016).

The rubber hand illusion is of specific relevance to human-machine embodied cooperation. It is one of the more encouraging pieces of evidence suggesting that non-bodily objects and even cooperative machines such as a robotic prosthesis (described below) can engage the brain's mechanisms that distinguish self or other. Yet here, the appropriateness of bidirectional human-machine communication becomes a key element. The rubber hand illusion is diminished in cases where visual and tactile stimulation are asynchronous, demonstrating that the congruency of multisensory inputs is vital to the illusion (Botvinick and Cohen, 1998). Therefore, for a cooperative machine to engage one's sense of ownership, the sensory feedback from that system must be strategically designed and tuned. The rubber hand illusion has found many applications throughout human-machine cooperative literature and purposefully developing a sense of ownership has been a goal in prosthetic limbs (discussed below) (Niedernhuber et al., 2018), chronic pain treatment (Martini, 2016), and virtual reality avatars (Matamala-Gomez et al., 2019).

### The Sense of Agency

The sense of agency is distinct from the sense of ownership and can be thought of as the feeling of “mineness” for our actions. It distinguishes our self-generated actions (and their outcomes) from those generated by others (David et al., 2008). It accounts for the experience of authoring our actions and is captured in statements such as “I moved my leg” or “I pressed the button and made that happen” (Jeannerod, 2003; Braun et al., 2018). The sense of agency emerges when the motor and sensory outcomes of our actions align with our brain's predictions of the body acting in its environment (internal models) (Gallagher, 2000; Wolpert et al., 2001; Van Den Bos and Jeannerod, 2002; Legaspi and Toyoizumi, 2019). There are two levels of agency (Wen, 2019), both of which have implications in cooperations with autonomous machines. The first emerges during the control of our bodies (internal-agency). As humans, we trust our bodies to perform the actions we intend; when this is achieved, we establish an intrinsic sense of agency that is closely coupled, yet distinct from the sense of ownership (Gallagher, 2000). This sense is largely influenced by the intentions and brain's predictive models of a movement as well as the sensory experiences generated in our bodies (Gallagher, 2000; Marasco et al., 2018). The second level describes the experience of controlling external events (external-agency) (Wen et al., 2019). Pressing buttons, pulling levers, and even operating complex machinery falls into this category (Wen et al., 2019). Internal- and external-agency are both highly relevant to the perception of authoring outcomes during human-machine cooperations. Importantly, it only forms when user actions and internal models align with the sensory information returned from the machine and environment, an important goal when designing a machine's bidirectional interface.

Promoting a sense of agency during autonomous human-machine cooperation is important as it allows the user to assume authorship over cooperative actions; and therefore, may promote more forgiving interactions. The sense of agency is heavily influenced by our perceptions of self or other, and when achieved, individuals will explicitly judge themselves as responsible for the outcomes of actions (Dewey and Knoblich, 2014; Braun et al., 2018; Schofield et al., 2019). Not only does it influence explicit perceptions, but also subconscious processes. When an action produces an appropriate sensory outcome, the action and outcome are perceived as closer together in time, a phenomenon known as intentional binding (Haggard et al., 2002). Of further relevance, the sense of agency may be formed during cooperative actions. In human-human cooperations, a joint sense of authorship may be formed (Obhi and Hall, 2011; Sahai et al., 2017). Yet, these effects are diminished if a human partner is replaced with a machine (Obhi and Hall, 2011; Sahai et al., 2017; Grynszpan et al., 2019), and increasing autonomy in machine-partners reduces the sense of agency (Berberian et al., 2012). Relevant to interactions with autonomous machines, we suggest that the communicative potential of cooperating with our own bodies, another human, or a machine is dramatically different and reflected in our brain's models of these partnerships. The sense of agency is important in achieving embodied cooperation, and cooperative machines have the potential to form a joint sense of agency (or even external or internal agency) through careful construction of bidirectional interfaces. Consistent and accurate contributions of the machine will be necessary, and relevant temporally-appropriate sensory feedback will be required to allow the brain to build robust internal models.

## Integrating Machines As a Part of Ourselves

There are numerous examples of bidirectional human-machine interfaces that promote embodied cooperation. Some of the more prominent work has emerged in the active field of advanced artificial limbs [for reviews see (Niedernhuber et al., 2018; Sensinger and Dosen, 2020)]. Robotic upper limb prostheses are computerized machines, and here embodiment may be an intuitive goal as they are often prescribed to augment or return function after limb loss. Like many other cooperative technologies, control and sensory feedback remain a driving factor influencing device abandonment (Biddiss and Chau, 2007; Østlie et al., 2012; Schofield et al., 2014). However, experimental prosthetic sensory interfaces that provide (most commonly) touch-based feedback have become widely investigated. Studies have shown that various modalities of feedback including vibration, skin-based pushing forces, and electrical stimulation of relevant nerves can be integrated into the brain's sensorimotor control loops and even promote a sense of ownership over an artificial limb (Ehrsson et al., 2008; D'Alonzo et al., 2015; Blustein et al., 2018; Graczyk et al., 2018; Valle et al., 2018; Cuberovic et al., 2019).

Recently, bidirectional neural-machine interfaces have been established for robotic prosthesis users. One such example leverages targeted reinnervation (TR) surgery to provide prosthetic control through users thinking about moving their missing limbs (Kuiken et al., 2004), and can even restore the senses of touch (Kuiken et al., 2007) and movement (Marasco et al., 2018). Working with individuals that received TR surgery, Marasco et al. used a modified version of the rubber hand illusion to demonstrate that ownership over a prosthesis can be readily achieved (Marasco et al., 2011). When participants viewed touch to a prosthetic hand while receiving synchronous sensations of touch to their missing hands, a strong sense of ownership was formed and captured across multiple independent measures. Since, Schofield et al. have reported on a long-term trial of similar touch-enabled prostheses (Schofield et al., 2020). Restoring touch sensation improved participants' grasping abilities, and over time participants tightly integrated touch into their prosthesis control strategies. Participants demonstrated long-term adaptations, developing a strong sense of ownership only when feedback was temporally and spatially appropriate (Schofield et al., 2020). Individuals who received TR surgery can also form an internal sense of agency over their prostheses. Vibration of muscles and/or tendons can induce illusory perceptions of limb movement (Goodwin et al., 1972), and vibration of participants' reinnervated muscles can induce perceptions of missing hand movements (Marasco et al., 2018). Marasco et al. demonstrated that these sensations of missing hand movement can be integrated with visual information of a prosthesis moving to influence perceptions of self-generated actions and develop a strong sense of internal-agency (Marasco et al., 2018).

Beyond these studies, prosthetic embodiment has been a rapidly growing area of interest. In fact, a PubMed search of the terms prosthetic, (or) prosthesis, (or) artificial limb, (and) embodiment, returned 195 research articles in the 30 years between 1989 and 2019. Nearly 80% of these articles were published in the last 10 years. Here, we are reaching a critical mass and beginning to reshape the way we view the relationship between a prosthesis and user. As robotic prostheses continue to advance, we are beginning to depart from simply evaluating these devices as tools for improved function and starting to assess their influence on the mechanisms that drive embodiment, an important next step.

## Discussion

The distinction of self or other shapes our perceptions of nearly every action we perform and drives our propensity to blame cooperative technologies when errors are made. Autonomous systems are becoming increasingly integrated into our society, and we need to reframe how we approach our cooperative relationships such that they engage the fundamental mechanisms that distinguish self or other. Neurally interfaced prostheses provide a strong example of how we may begin achieving this goal; however, they are far from the only technology in which embodied cooperation is desirable. Other assistive devices such as orthotic exoskeletons and powered mobility aids may also benefit. In these applications, the goal of bidirectional interfaces may be two-fold: the first being effective control to improve the user's physical capabilities, and the second being the embodiment of the technology. If such devices are truly embodied, the capabilities they afford the user become perceived as body function. This is a significant shift for the user as they depart from feelings of dependance on a machine to feelings of being more independent and physically capable with their bodies. It is important to note that ethical considerations will grow increasingly important as we move closer to seamless partnerships and even begin to augment human capabilities. We will need to be cognizant of the relationships and dependencies we create with machines; their implications to the user and society; as well as their accessibility and equity, especially in medical care contexts; among many others.

Full embodiment of every cooperative machine is an incredibly ambitious goal. However, as our society and our relationships with autonomous machines continues to evolve, cooperative embodiment may provide meaningful pathways to promote effective control, foster forgiving interactions, and encourage device adoption. The experience of embodiment arises from the senses of self-location, ownership, and agency, all of which cross a spectrum of workspaces that may be targeted by various cooperative machines. Just as it is valuable in prostheses, we will need to begin shifting how we evaluate interactions with cooperative machines to include assessments of cooperative embodiment. In doing so we can begin carefully constructing contextually appropriate bidirectional interfaces that leverage our inborn distinctions of self or other, and flip our natural biases to accept cooperative machines and their actions as indistinguishable from our own.

## Data Availability Statement

The original contributions presented in the study are included in the article/supplementary material, further inquiries can be directed to the corresponding author/s.

## Author Contributions

JS participated in the preparation of the figure. PM participated in writing, figure generation, and shaping the scientific direction of the manuscript. All authors contributed to the writing, reviewing, and editing processes as well as figure generation.

## Conflict of Interest

The authors declare that the research was conducted in the absence of any commercial or financial relationships that could be construed as a potential conflict of interest.
